# Perioperative acute myocardial infarction rate in chronic renal disease patients undergoing orthopedic surgery: Is there any difference between dialyzed and nondialyzed patients?

**DOI:** 10.1371/journal.pone.0210554

**Published:** 2019-01-17

**Authors:** Thung-Lip Lee, Feng-Chen Kao, Yao-Chun Hsu, Ying-Ying Lo, Yuan-Kun Tu

**Affiliations:** 1 Department of Cardiovascular, E-Da Hospital, Kaohsiung, Taiwan; 2 School of Medicine, I-Shou University, Kaohsiung, Taiwan; 3 Department of Orthopaedics, E-Da Hospital, Kaohsiung, Taiwan; 4 School of Medicine, Fu-Jen Catholic University, New Taipei, Taiwan; 5 Department of Internal Medicine, E-Da Hospital, Kaohsiung, Taiwan; 6 Graduate Institute of Clinical Medical Science, China Medical University, Taichung, Taiwan; 7 Department of Healthcare Administration, I-Shou University, Kaohsiung, Taiwan; Thomas Jefferson University, UNITED STATES

## Abstract

**Background:**

The incidence of acute myocardial infarction (AMI) in healthy patients undergoing noncardiac surgery is <1%. When patients with chronic kidney disease (CKD) undergo orthopedic surgery, AMI incidence can be expected to be relatively high. However, data on a population-wide scale is lacking.

**Objective:**

To investigate AMI incidence in patients with CKD (with and without dialysis) undergoing orthopedic surgery.

**Design:**

A population-based study covering the period from January 1, 1997, to December 31, 2011.

**Setting:**

Data from the Taiwan National Health Insurance Research Database.

**Participants:**

Participants were 219,195 patients with CKD who underwent surgery between January 1, 1997, and December 31, 2011.

**Results:**

AMI occurred in 2,708 participants (1.24%). The AMI incidence rate in the dialyzed group was 1.52%, which was higher than that in the nondialyzed group after propensity score matching. Dialysis (odds ratio [OR]: 1.79; 95% confidence interval [CI]: 1.62–1.98), male (OR: 1.42; 95% CI: 1.28–1.57), diabetes mellitus (OR: 1.61; 95% CI: 1.44–1.80), hyperlipidemia (OR: 1.88; 95% CI: 1.68–2.11), old myocardial infarction (OR: 18.87; 95% CI: 16.26–1.21.90), and cerebral vascular disease (CVA) (OR: 1.29; 95% CI: 1.30–1.47) were all associated with AMI in the patients with CKD.

**Conclusions:**

The AMI risk was higher in the patients with CKD undergoing orthopedic surgery than in the general population, and the dialyzed group had a higher risk of AMI than did the nondialyzed group.

## Introduction

Acute myocardial infarction (AMI), commonly known as heart attack, is a major cause of morbidity and mortality worldwide. Typical symptoms include sudden chest pain (typically radiating to the left arm or left side of the neck), shortness of breath, nausea, vomiting, palpitations, sweating, and anxiety (often described as a sense of impending doom) [1]. Women may experience fewer typical symptoms than men, most commonly shortness of breath, weakness, a feeling of indigestion, and fatigue [2]. Approximately a quarter of all AMI cases are “silent” (without chest pain or other symptoms). When AMI occurs in patients undergoing surgery, symptoms are similarly “silent” and difficult to detect. Despite advances in medical technology, cardiovascular complications, such as AMI, are still a major contributor to mortality after noncardiac surgery [3].

The incidence of AMI considerably differs between patients undergoing noncardiac surgery and healthy people. In healthy people, the incidence rate of AMI is <1% [4–7]. In patients with heart disease and multiple risk factors for cardiovascular disease, the incidence of perioperative AMI ranges from 3% to 17% [7–12]. The incidence of silent myocardial infarction in patients having a high risk of coronary artery disease (CAD) or ischemic complications during orthopedic surgery is similar to that during vascular surgery (30%–40%) [13, 14].

Chronic kidney disease (CKD), defined as an estimated glomerular filtration rate (eGFR) of <60 mL/min/1.73 m2, is associated with increased AMI [15] and cardiovascular mortality. End-stage renal disease (ESRD) is defined as stage 5 CKD with eGFR < 15 mL/min/1.73 m2 and normally requires dialysis treatment or a kidney transplant.

CKD and traditional risk factors for CAD contribute to a high prevalence of CAD. Patients with CKD also have a higher incidence of AMI and higher all-cause cardiovascular mortality than healthy people. In ESRD, CAD accounts for 38%–40% of deaths in patients beginning dialysis. AMI is related to high mortality rates in patients on both short- and long-term dialysis [16].

CKD is an independent risk factor for cardiovascular events and death after noncardiac surgery [17, 18]. In modern medicine, the practice of orthopedic surgery is increasing. When patients with CKD (including ESRD) undergo orthopedic surgery, the incidence of AMI can be expected to be relatively high.

In this population-based study, we investigated the incidence of AMI in patients with CKD undergoing orthopedic surgery over a 15-year period. In addition, we evaluated whether the incidence of AMI differs between CKD patients undergoing dialysis and those not undergoing dialysis.

## Materials and methods

### Study design and data source

We analyzed data from Taiwan’s National Health Insurance Research Database (NHIRD) in this retrospective study. The Ministry of Health and Welfare launched the National Health Insurance (NHI) program in 1995 to cover the health care of 22.9 million residents of Taiwan. The NHI is a single-payer system with a compulsory universal policy that covers more than 99% of the national population. The NHIRD comprises a comprehensive registry of claims data for research purposes and is maintained by the Taiwan National Health Research Institute (NHRI). In the NHIRD, diseases are coded according to the International Classification of Diseases, 9th Revision, Clinical Modification (ICD-9-CM) criteria. Numerous studies have demonstrated the utility of the NHIRD in population-based research [10, 11]. Data acquisition for this study was approved by the NHRI, and the study protocol was certified as exempt from review by the Institutional Review Board of E-Da Hospital, whereas the study itself did not require full review board approval.

### Patient selection and definition

We analyzed the NHRID to identify study patients and selected all data for adult inpatients with CKD between January 1, 1997, and December 31, 2011. Patients with CKD were identified by the presence of the following ICD-9-CM codes in their records: 250.4 (diabetes with renal manifestations), 274.1 (gouty nephropathy), 403 (hypertensive chronic kidney disease), 404 (hypertensive heart and chronic kidney disease), 440.1 (disease of renal artery), 442.1 (disease of renal artery), 447.3 (hyperplasia of renal artery), 581 (nephritic syndrome), 583 (nephritis and nephropathy), 585 (chronic renal failure), and 588 (disorders resulting from impaired renal function). Patients whose records contained ICD-9-CM codes 283.1 (hemolytic uremic syndrome), 572.4 (hepatorenal syndrome), 42.1 (hypertension secondary to renal disease, complicating pregnancy, childbirth, and the puerperium), 646.2 (unspecified renal disease in pregnancy, without mention of hypertension), and V594 or V420 (transplant) were excluded. Patients with ESRD were defined as those with type 4 major injuries and with the code 58001C (dialysis therapy). Orthopedic surgeries included in this study were total joint replacement surgeries (64164B and 64162B), hip surgeries (64029B and 64170B), and posterior spine surgeries (83002C, 83003C, 83045B, 83046B, 64225B, and 64226B).

We selected patients with CKD who received orthopedic surgical treatment according to anesthesia code numbers 96017C, 96020C, 96007C, and 96005C. Patients who received only coronary artery bypass graft surgery during their admission period were excluded. The records of all hospitalizations provided a substantial amount of information. We linked study patients with inpatient claims data to identify episodes of AMI (ICD-9-CM 410.ab; a = 0–9; b = 0 or 1) in patients during hospitalization. We further classified cases into two groups—dialyzed and nondialyzed—to evaluate any differences in the incidence of AMI between them. Both the groups were matched by propensity scores generated through logistic regression built on the following baseline characteristics affecting AMI risk: age, sex, hypertension, diabetes mellitus, cerebrovascular accident (CVA), hyperlipidemia, peripheral artery occlusive disease (PAD), and a history of old MI (myocardial infarction (MI).

### Ascertaining demographic and comorbid variables

We included patient demographics, mortality, and baseline comorbidities, namely hypertension, diabetes mellitus, CVA, hyperlipidemia, PAD, and a history of old MI.

### Statistical analysis

The incidence rate of AMI was calculated as the number of cases of AMI per 100 person-years. The association between dialysis and AMI was examined using a Cox proportional hazard model that took age, sex, and baseline comorbidities into account. Univariate and multivariate adjusted hazard ratios were recorded. Calculated results are expressed as point estimates along with 95% confidence intervals (CIs). All statistical tests with a *P* value less than 0.05 were defined as significant. All data management and calculation were performed using SPSS Statistics for Windows version 24.0 (SPSS, Inc., Chicago, IL, USA).

## Results

### Baseline characteristics of the study population

A total of 219,195 patients with CKD who underwent orthopedic surgery under general, spinal, or epidural anesthesia were enrolled in the study. Among them, 106,259 (48.5%) were ESRD cases.

**Fig 1:**
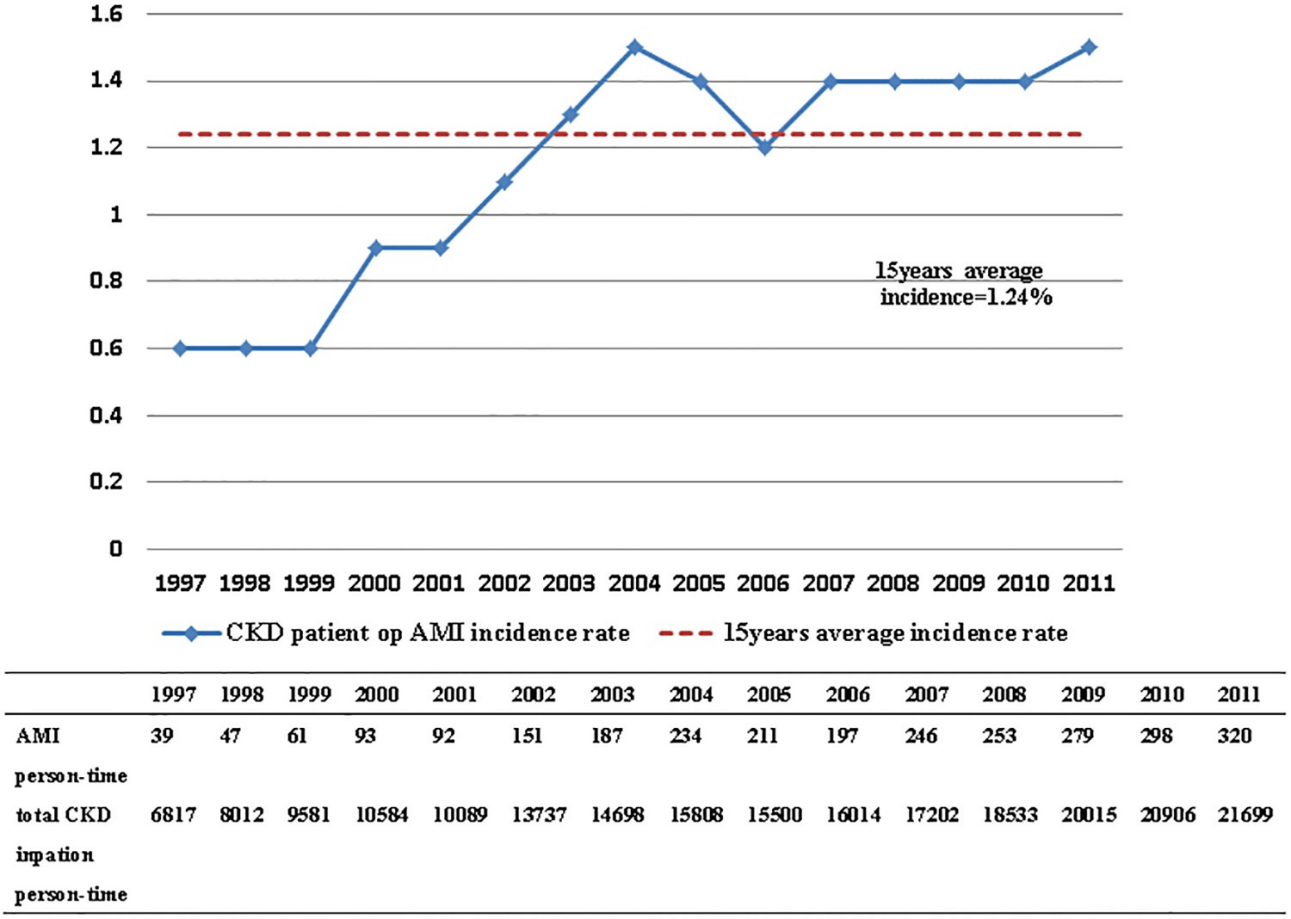
The rate of AMI in all CKD Subjects received surgeries (N = 219195) in 15 years. AMI (Acute myocardial infarction); CKD (chronic kidney disease).

**Fig 2:**
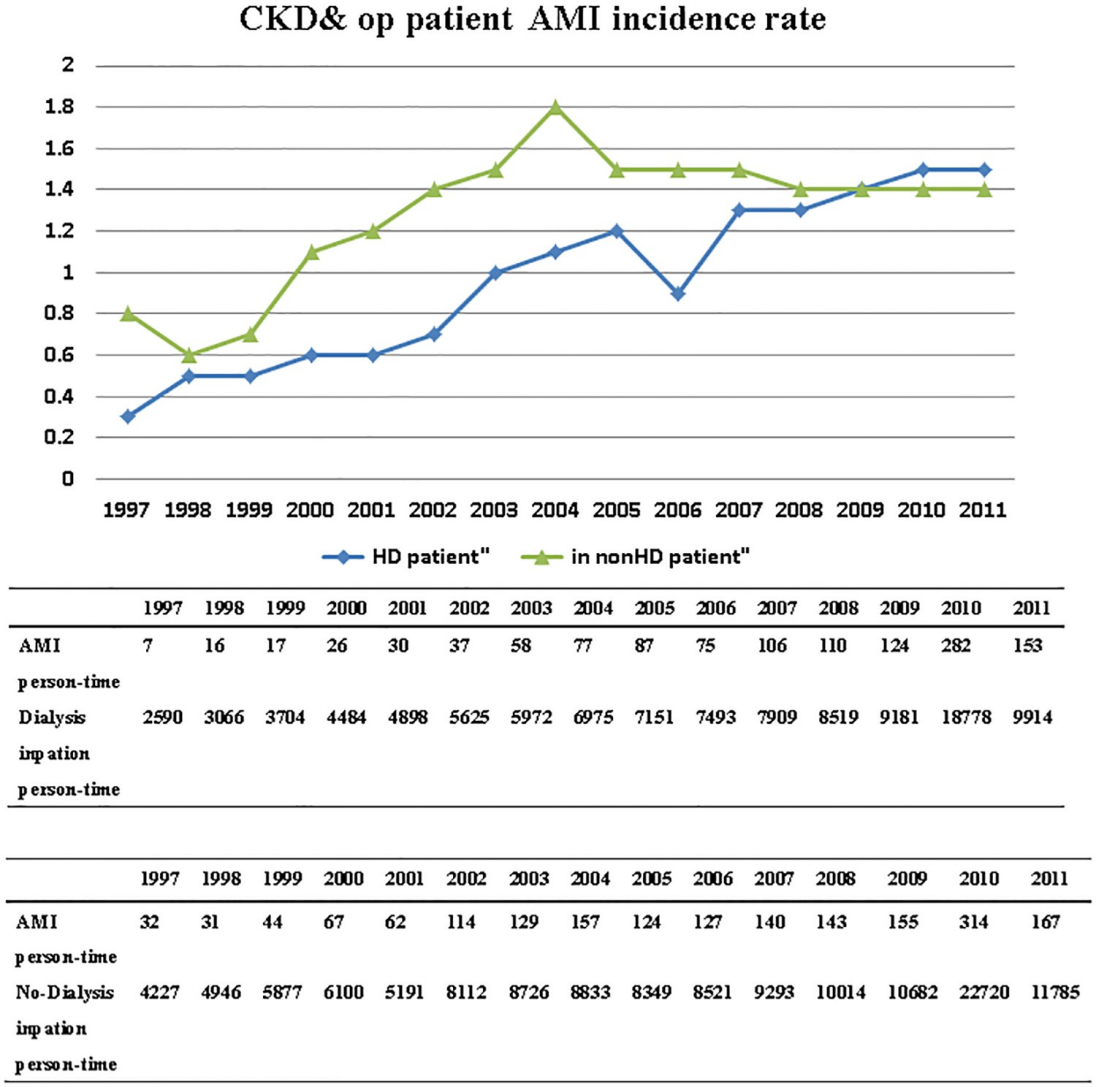
The rate of AMI in CKD (with/ without dialysis) cases with surgery in 15 years. AMI (Acute myocardial infarction); CKD (chronic kidney disease); op (operation).

AMI occurred in 2,708 (1.24%) of the participants. The annual incidence rate of AMI for all the patients with CKD undergoing orthopedic surgery is listed in Fig 1. The number of patients with CKD requiring inpatient orthopedic surgery increased from 6,817 in 1997 to 21,699 in 2011. The overall incidence of AMI in this population also increased from 0.57% in 1997 to 1.47% in 2011. The annual incidence rates of AMI of CKD patients both with and without dialysis after orthopedic surgery is listed in Fig 2. The average AMI incidence of patients with CKD was 1.52% in the dialyzed group and 1.4% in the nondialyzed group. Both the groups exhibited an increasing trend of AMI incidence from 1997 to 2011.

### Case–control study

Among the 219,195 CKD patients analyzed, case–control groups were created after propensity score matching, with a total of 66,828 CKD cases. There were 26,802 and 40,026 male and female patients, respectively, whose average age was 70.59 years (Table 1). After matching, the dialyzed group had a higher AMI rate (3.51%) than did the nondialyzed group (2.12%; *P* < 0.0001), and the mortality rate was also higher in the dialyzed group (1.47% vs. 0.42%; *P* < 0.001).

**Table 1:** Characteristics of CKD Subjects with surgeries (N = 66,828) in the case-control study (matched).

	Non-dialysis N = 44,552	Dialysis N = 22,276	p-value
Age	70.59±11.54	70.59±11.54	0.9939
Age Group			>.9999
<50	2494(5.60)	1247(5.60)	
50–64	9212(20.68)	4606(20.68)	
65–79	24034(53.95)	12017(53.95)	
> = 80	8812(19.78)	4406(19.78)	
Gender			>.9999
Female	26684(59.89)	13342(59.89)	
Male	17868(40.11)	8934(40.11)	
Comorbidities			
Diabetes	21258(47.72)	10629(47.72)	>.9999
Hypertension	21315(47.84)	10724(48.14)	0.4665
Hyperlipidemia	5389(12.1)	2662(11.95)	0.5849
PAOD	6(0.01)	1(0.00)	0.2850
CVA	4883(10.96)	2474(11.11)	0.5700
Old MI	672(1.51)	331(1.49)	0.8220
CABG	15(0.03)	18(0.08)	0.0097
PCI	49(0.11)	49(0.22)	0.0005
AMI	943(2.12)	783(3.51)	<.0001
Mortality	189(0.42)	327(1.47)	<0.0001

### Multivariate adjusted evaluation of the difference in AMI incidence between dialyzed and nondialyzed groups

According to the results of multivariate analysis, the dialyzed group was found to have a higher risk of AMI (odds ratio [OR]: 1.79: 95% CI: 1.62–1.98). The results also indicated that male sex (OR: 1.42; 95% CI: 1.28–1.57), diabetes mellitus (OR: 1.61; 95% CI: 1.44–1.80), hyperlipidemia (OR: 2.70; 95% CI: 2.41–3.02), hypertension (OR 1.88; 95% CI: 1.68–2.11), CVA (OR: 1.29; 95% CI: 1.13–1.47), and a history of old MI (OR: 18.87; 95% CI: 16.26–21.90) were associated with AMI in CKD patients (Table 2). Adjustment for PAD could not be performed because of its low incidence in the control group.

**Table 2:** Multivariate adjusted evaluation of the difference in AMI incidence between dialyzed and nondialyzed groups.

	Crude		Adjusted	
	OR(95%CI)	p-value	OR(95%CI)	p-value
Dialysis	1.68(1.53–1.85)	<.0001	1.79(1.62–1.98)	<.0001
Male vs. Female	1.37(1.25–1.51)	<.0001	1.42(1.28–1.57)	<.0001
Comorbidities				
Diabetes	2.31(2.09–2.56)	<.0001	1.61(1.44–1.80)	<.0001
Hypertension	2.71(2.44–3.00)	<.0001	1.88(1.68–2.11)	<.0001
Hyperlipidemia	4.02(3.63–4.46)	<.0001	2.70(2.41–3.02)	<.0001
PAOD	NA		NA	
CVA	2.05(1.82–2.32)	<.0001	1.29(1.13–1.47)	0.0001
Old MI	24.72(21.48–28.45)	<.0001	18.87(16.26–21.90)	<.0001

## Discussion

A community-based study indicated that age- and sex-adjusted AMI incidence in the United States increased from 274 cases per 100,000 person-years in 1999 to 287 cases per 100,000 person-years in 2000, decreasing each year thereafter to 208 cases per 100,000 person-years in 2008 [19]. In Taiwan, the incidence of AMI continued to increase over these study periods. According to a population cohort study, the incidence of AMI in the healthy population in Taiwan increased from 1997 to 2011, with age- and sex-adjusted incidence rates (per 100,000 people) rising from 30 in 1997 to 42 in 2011 [20]. Another study demonstrated the same increasing trend of age-adjusted incidence rates (per 100,000 people), from 28 in 1999 to 44.4 in 2008 [21].

The incidence rate of acute coronary syndrome (ACS) in dialysis patients was higher in the Taiwanese Chinese population than in the general population. In an analysis of 19,974 adult patients using the NHIRD, Chou [22] reported the AMI incidence rate among dialysis patients to be approximately 4.1%. In our study, the average perioperative AMI incidence was 1.24% in the patients with CKD undergoing surgery, which was higher than that in the healthy population and consistent with that reported in other studies. The AMI incidence in dialyzed patients after surgery was approximately 1.52% in 2011, whereas it was 1.41% in nondialyzed group. As shown in Figs 1 and 2, an increasing number of patients with CKD required inpatient orthopedic surgical treatment and the AMI incidence continually increased, although never exceeding 1.6%.

After propensity score matching, the AMI rate in the dialyzed group was 3.51%, which was higher than that in the nondialyzed group (2.58%). The results of multivariate analysis also demonstrated that dialysis contributed to AMI events (OR: 1.79; CI: 1.62–1.98; *P* < 0.001). This finding is consistent with that of a meta-analysis conducted by Vashistha who reported that AMI risk increases with a decrease in eGFR. He reported that a increase in AMI risk as the eGFR declined from 30 to 60 (CKD group) to <30 ml/min/1.73 m^2^ (ESRD) [15].

The results of multivariate analysis in our study indicated that male sex, hypertension, diabetes, hyperlipidemia, cerebral vascular disease, and a history of old MI were all related to AMI risk. Risk factors for CKD are similar to those for cardiovascular disease, including hypertension and diabetes [22]. Patients on chronic dialysis have a high incidence of atherosclerotic coronary disease associated with an extremely high risk of ACS upon dialysis initiation [23]. AMI is the leading cause of mortality in patients undergoing dialysis, accounting for approximately 40% of all deaths [24]. Patients maintained on chronic dialysis also have a high burden of atherosclerotic coronary disease associated with an extremely high risk of ACS [25]. CAD, CVA, PAD, and heart failure have also been found to increase AMI risk following orthopedic surgery [26].

Our study demonstrated that AMI risk was higher in the dialyzed group than in the nondialyzed group of patients with CKD after orthopedic surgery. The higher AMI rate in the dialyzed group can be attributed to several reasons. Prior cardiovascular disease, a known risk factor for cardiovascular events and death, is more frequent in patients with lower eGFR levels. Reduced kidney function is also associated with increased inflammatory factors, abnormal apolipoprotein levels, elevated plasma homocysteine levels, enhanced coagulability, anemia, left ventricular hypertrophy, increased arterial calcification, endothelial dysfunction, and arterial stiffness [27].

Go et al. reported that worsening renal function had an independent inversely graded correlation with all-cause mortality [28]. Patients with CKD, particularly those on maintenance dialysis, have poor outcomes after the occurrence of ACS [29–33]. Chou [22] reported an overall in-hospital mortality rate of 9.7% in the general population. Herzog et al. [34] reported that mortality rates for dialyzed patients with AMI were 26% in 1998 and 21.6% in 2007, which were higher than that in the general population. Our study also demonstrated a higher mortality rate in the dialyzed group after orthopedic surgery.

In summary, we observed an increasing incidence of perioperative AMI in patients with CKD receiving orthopedic surgery in Taiwan. In the patients who underwent orthopedic surgical treatments, AMI rate and post-AMI mortality rates were higher in the dialyzed group than in the nondialyzed group. This AMI risk should be recognized to avoid potential adverse effects in perioperative status and ensure patient safety.

Our study has some limitations. First, the disease diagnosis relied on claims data and ICD-9-CM codes, which could result in misclassification. Asymptomatic AMI according to the new definition was unable to detect in our study and the actual AMI events may not always be identified in the NHIRD. However, diagnostic codes for AMI and CKD used in the NHIRD have been validated by other studies [35–36]. Second, we were unable to calculate eGFRs or determine CKD stages from retrospective claims data. Third, this study also lacked specific data on dialysis adequacy, laboratory results, and medical prescriptions, all of which could affect AMI.
